# A systematic quantification of carbonic anhydrase transcripts in the mouse digestive system

**DOI:** 10.1186/1471-2199-8-22

**Published:** 2007-03-16

**Authors:** Pei-wen Pan, Alejandra Rodriguez, Seppo Parkkila

**Affiliations:** 1 Institute of Medical Technology, University of Tampere and Tampere University Hospital, 33520, Tampere, Finland

## Abstract

**Background:**

Carbonic anhydrases (CAs) are physiologically important enzymes which participate in many gastrointestinal processes such as acid and bicarbonate secretion and metabolic pathways including gluconeogenesis and ureagenesis. The genomic data suggests that there are thirteen enzymatically active members of the mammalian CA isozyme family. In the present study, we systematically examined the mRNA expression levels of all known CA isozymes by quantitative real-time PCR in eight tissues of the digestive system of male and female mice.

**Results:**

The CAs expressed in all tissues were Car5b, Car7, and Car15, among which Car5b showed moderate and Car7 and Car15 extremely low expression levels. Car3, Car12, Car13, and Car14 were detected in seven out of eight tissues and Car2 and Car4 were expressed in six tissues. Importantly, Car1, Car3, and Car13 showed very high expression levels in certain tissues as compared to the other CAs, suggesting that these low activity isozymes may also participate in physiological processes other than CA catalysis and high expression levels are required to fulfil their functions in the body.

**Conclusion:**

A comprehensive mRNA expression profile of the 13 enzymatically active CAs in the murine gastrointestinal tract was produced in the present study. It contributes to a deeper understanding of the distribution of CA isozymes and their potential roles in the mouse digestive system.

## Background

Mammalian α-carbonic anhydrases (CAs) are a large enzyme family containing 16 different isoforms, among which 13 (CA I, II, III, IV, VA, VB, VI, VII, IX, XII, XIII, XIV, and XV) are enzymatically active. The other three carbonic anhydrase-related proteins (CA-RP VIII, X, and XI) appear to lack CA activity because of substitutions in one or more of the functionally important histidine residues. In addition, the receptor-type protein-tyrosine phosphatases (RPTP) β and γ have been reported to contain 'CA-like' domains [1-3]. The enzymatically active CAs catalyze the reversible hydration of carbon dioxide in the reaction CO_2 _+ H_2_O ⇔ HCO_3_^- ^+ H^+^, and participate in various biological processes, including CO2 transport, regulation of pH homeostasis, bone resorption, ureagenesis, gluconeogenesis, production of body fluids, and fertilization [1,4]. Each isozyme also has a characteristic subcellular localization. CA I, II, III, VII, and XIII are cytosolic, CA IV, IX, XII, XIV, and XV are membrane-associated, CA VA and VB are mitochondrial, and CA VI is secreted. According to a recent bioinformatic analysis, CA XV may be the last isoform of the mammalian CA gene family to be identified [5]. The identification of all isoforms of this gene family opens up a new avenue for studying the expression and function of all CAs in different tissues.

CAs have been found to be widely expressed along the entire gastrointestinal canal and in the main digestive glands where they participate in proton and bicarbonate secretion, detoxification of ammonia, production of pancreatic juice, as well as in absorption of salt and water in the intestine [6,7]. However, no systematic study has been conducted to date to describe the comprehensive expression of all known active CA isozymes in that region. In order to gain a deeper understanding of the distribution of CA isozymes and their potential role in the mouse digestive system, we used quantitative real-time PCR to assess the mRNA expression levels of Car1, Car2, Car3, Car4, Car5a, Car5b, Car6, Car7, Car9, Car12, Car13, Car14, and Car15 in the pancreas, liver, oesophagus, stomach, duodenum, jejunum, ileum, and colon of male and female mice.

## Results

### Expression of transcripts for cytosolic CA isozymes

**Figure 1:**
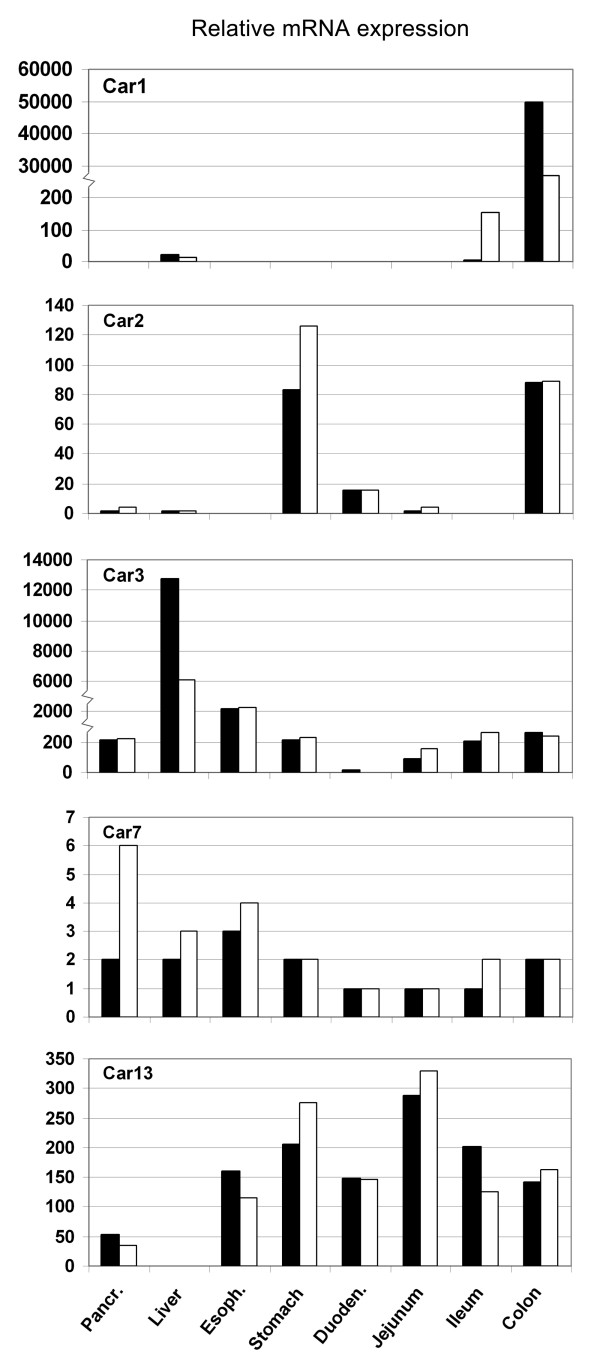
**Expression of transcripts for cytosolic CAs**. Solid and open bars represent results obtained from male and female mice, respectively.

In this study we investigated the mRNA expression of all five active cytosolic CA isozymes by quantitative real-time PCR in the mouse digestive system (Fig. 1). Car1 had a narrow expression profile and was detected in three out of eight tissues, with a very high signal in the colon. Lower expression was also detected in the liver. Differences in male and female expression levels of Car1 were observed in the ileum and colon. In the ileum, the expression level was much higher in female mice than in males, whereas the opposite was true in the colon, where the expression level in male mice was almost twice as high as in the female mice.

Car2 mRNA expression was found in the pancreas, liver, stomach, duodenum, jejunum, and colon. It was most intense in the stomach and colon, moderately intense in the duodenum, and present at lower levels in the pancreas, liver, and jejunum. No marked sex differences were found in the expression levels.

Surprisingly, Car3 had an extremely high expression level in all tissues except for the duodenum, as compared to the other CA transcripts. Its expression was highest in the liver, followed by the oesophagus, colon, and other positive tissues. Furthermore, its expression level in the liver of male mice was about twice as high as in females, while no apparent difference was found in other tissues.

Car7 showed a broad expression pattern, being present in virtually all of the tissues studied, albeit at a very low level. Although expression levels in the pancreas differed between male and female mice, we would expect no significant functional consequences from this difference given the low expression of this transcript.

Car13 was another highly expressed transcript in all tissues except for the liver. Its highest expression was found in the stomach and jejunum, followed by the oesophagus, duodenum, ileum, colon, and pancreas. No significant difference was observed in the expression levels in male and female mice.

### Expression of transcripts for membrane-associated CA isozymes

**Figure 2:**
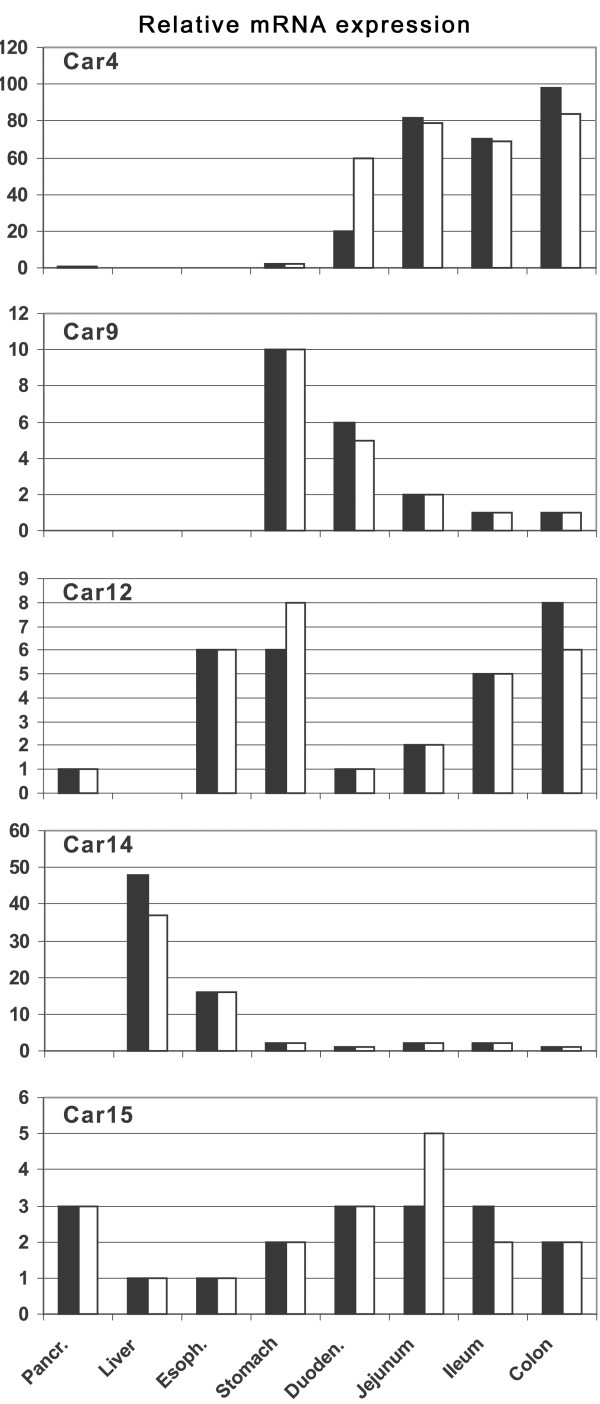
**Expression of transcripts for membrane-associated CAs**. Solid and open bars represent results obtained from male and female mice, respectively.

As shown in Figure 2, the expression of different membrane-associated CAs varied dramatically along the alimentary tract. The Car4 expression level increased from no or a very low signal in the oesophagus and stomach to a strong one in the colon. The only gender difference was observed in the duodenum, where the signal was three times higher in the female mice compared to the males. Very low Car4 expression was also detected in the pancreas. As expected based on a previous study [8], Car9 showed the highest signal in the stomach, and the expression decreased in the more distal segments of the gastrointestinal canal. Car9 expression was below the detection limit in the liver, pancreas, and oesophagus.

Car14 was most abundant in the liver, moderately expressed in the oesophagus, and barely detectable in other tissues. The highest Car12 mRNA levels were detected in the oesophagus, stomach, ileum, and colon. In the gut, the expression of Car12 mRNA increased along the cranial-caudal axis, being most prominent in the colon. Car15, which has been identified most recently, was expressed at very low levels in all the tissues studied. The expression levels were just above the detection limit of the quantitative PCR.

### Expression of transcripts for mitochondrial and secretory CA isozymes

**Figure 3:**
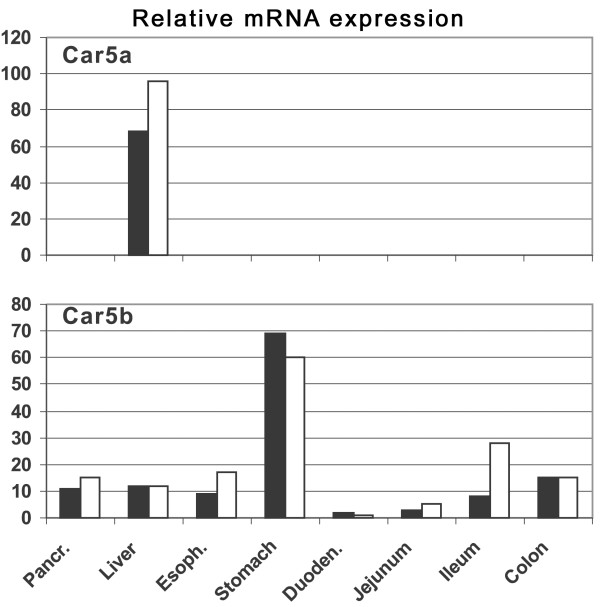
**Expression of transcripts for mitochondrial CAs**. Solid and open bars represent results obtained from male and female mice, respectively.

The results presented in Fig. 3 indicate that the mitochondrial Car5a was found only in the liver, as expected [9], and no difference was detected between male and female mice. Car5b, on the other hand, had a wider expression pattern. It was observed in all tissues examined in the present study, with the highest signal detected in the stomach. Car5b expression was low in the pancreas, liver, oesophagus, ileum, and colon. The tissues showing the lowest signal were the duodenum and jejunum. Male and female mice showed quite similar expression levels, except for the ileum where females showed a markedly higher signal. Car6 was the only CA transcript which was negative in all the tissues studied (data not shown).

## Discussion

**Figure 4:**
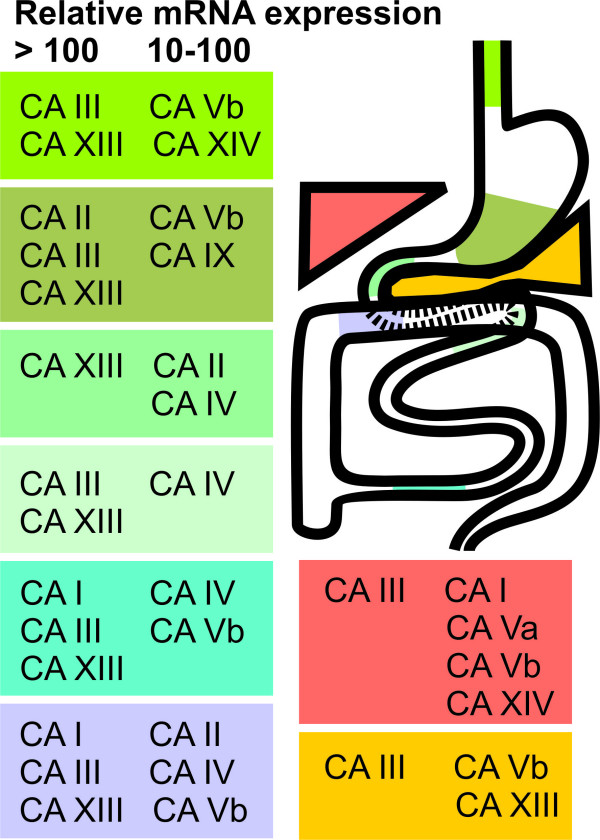
**A schematic model showing relative mRNA expression levels of different CA isozymes in the gastrointestinal tract**. Only the Car transcripts with moderate (relative expression value 10–100) or high expression (relative expression value >100) levels are included in the model.

The expression of individual CA isozymes in mouse tissues has been investigated in several previous studies by means of RT-PCR, northern blot, and immunohistochemical staining [3,8-12]. Although these studies have significantly contributed to our understanding of the distribution and role of CA isozymes, only one or just a few isozymes have been examined in each study. The recent identification of CA XV [5], most probably the last member of the mammalian CA gene family, has made possible the parallel examination of all CA isozymes, which was realized in practise for the first time during the present investigations. The results obtained clearly confirmed that each gastrointestinal organ or segment contains more than one CA isozyme (Fig. 4). A remarkable finding was also that all CA isozymes except for Car6 showed at least weak mRNA expression in some tissues studied. In some cases, the expression appeared to be generally very low as it was with Car7, Car9, Car12, and Car15. This finding should not, however, diminish the significance of these isozymes, because even low mRNA expression can contribute to an important physiological role. This can be easily realized from the distinct gastric phenotype which has been reported in CA IX deficient mice [13].

One surprising finding was that among all cytosolic isozymes, Car3 and Car13 showed the widest expression profiles. High levels of Car3 transcript were detected in the pancreas, liver, oesophagus, stomach, jejunum, ileum, and colon as compared to the other isozymes. Expression levels were very high in the liver and oesophagus (the latter containing substantial amounts of skeletal muscle) that agrees well with the results obtained in an earlier study [14]. The wide pattern of Car3 expression was quite surprising in the context of recent findings showing that Car3 may be dispensable for mice living in a standard laboratory environment and no gastrointestinal symptoms were reported in Car3^-/- ^mice [15]. It is conceivable that Car3 is functionally more important in abnormal conditions such as during oxidative stress [16] or starvation [17]. Interestingly, the expression level of Car3 transcript in the liver of male mice was twice as high as in females. This supports the notion that Car3 is hormonally regulated, as demonstrated in previous studies [18,19]. Similarly to Car3, Car13 was found to be highly expressed in the digestive tract in comparison with other CAs. Its mRNA was detected in the pancreas, oesophagus, stomach, duodenum, jejunum, ileum, and colon. The absence of Car13 in the mouse liver is in good agreement with previous findings [3]. Although our recent studies have shown that CA XIII exhibits low catalytic activity [3], the wide distribution of Car13 at high levels suggests that it may play an important role in these tissues.

To date, only a few studies have been performed to explore the expression of CA VII. Its mRNA has been reported to be abundant in the human salivary gland and rodent lung and brain, and it was predicted to be widely distributed, albeit at low levels [20,21]. Indeed, Car7 mRNA expression was observed in all eight tissues in our study, and the quantification of mRNA indicated very low signals in all of them. Even though previous studies have suggested that CA VII represents the most conserved and ancient form of CAs [22], it is rather difficult to explain the function of CA VII due to its low catalytic activity and limited expression pattern.

Analysis of the mRNAs for membrane-associated isozymes revealed high expression levels for Car4 and Car14 in the gut and liver, respectively. The increasing expression of Car4 towards the distal end of the gastrointestinal canal is consistent with previous findings in rabbit [23] and rat [24] and is also in line with the suggestion that CA IV participates in ion and fluid transport processes taking place in the distal small and large intestine [24]. A gender difference in Car4 expression was found in the duodenum: female mice had three times higher signal level than males. This finding suggests that Car4 expression might be directly or indirectly regulated by hormonal factors which remain to be explored in future studies.

Car15, a close homolog of Car4, has been recently characterized and found to be expressed in many species, including the mouse. Unlike all other CA isozymes, CA XV is not expressed in humans and chimpanzees [5]. Its mRNA was reported to be found in the mouse kidney, brain, testis, 7-day embryo, and 17-day embryo by conventional RT-PCR. Our present real-time PCR data showed positive expression in the liver, stomach, duodenum, jejunum, ileum, and colon, albeit at extremely low levels.

CA IX has shown a fairly restricted expression profile in normal mammalian tissues, whereas it is present in several human carcinomas [25]. Its expression has been investigated in the normal human, rat, and mouse gastrointestinal tracts [8,26,27]. In this study, Car9 showed the highest expression in the stomach, and the signal intensity decreased along with the cranial-caudal axis of the lower gastrointestinal canal, being barely detectable in the colon. Interestingly, in our previous study the expression of Car9 in the stomach of Car2^-/- ^mice compared to wild type mice was twice as high at the mRNA level and three times as high at the protein level, which may reflect compensatory upregulation of Car9 in response to the loss of the high activity of Car2 [12].

The second isozyme associated with cancer, CA XII, has been found mainly in the kidney and colon, while trace expression of its mRNA has also been detected in the rabbit duodenum, jejunum, and ileum [10,23,28]. Our data confirm these findings in the duodenum, jejunum, ileum, and colon. Surprisingly, Car12 mRNA was observed in the oesophagus and stomach at a comparable level to the colon, and trace expression was also found in the pancreas. Notably, the signals for Car12 were generally very low in all gastrointestinal tissues. This finding agrees well with our recent results which showed low expression in the stomach and colon and markedly higher signals in the kidney [12].

CA VA and CA VB are distinct isoforms in that they are localized to the mitochondrial matrix. CA VA expression is abundant in the liver, while CA VB is more widely distributed, being present in almost all tissues [9,29]. The same kind of expression pattern was observed in the present study: Car5a transcript was detectable only in the liver, whereas Car5b was present in all eight tissues studied. The differences in the distribution might reflect different physiological roles or efficacy for CA VA and VB in metabolic pathways requiring mitochondrial bicarbonate production.

## Conclusion

The present study provides the first comprehensive mRNA expression profile of the 13 enzymatically active CAs in the mouse digestive system. The present evidence that mRNAs for all CA isozymes except for CA VI are expressed in the gastrointestinal tissues clearly indicates the complexity of the physiological processes in this region. In addition, CA isozymes are known to be differentially expressed not only along the intestinal segments but also between different cell types within a particular organ. Finally, it is a matter of debate whether the regulatory mechanisms could be different depending on the gender and species. Even though some of these isozymes have heretofore been demonstrated only in rodents, it is conceivable that general transport and pH regulation mechanisms are qualitatively quite similar in various species [30,31]. Nonetheless, the described complex distribution of various Car mRNAs emphasizes the need for functional studies where certain CA isozymes are selectively targeted one at a time. To date only single and double knockout studies have been performed on CA isozymes [13,15,32,33], and making triple knockouts would be even more challenging. Isozyme-specific inhibitors, when available, would potentially offer novel tools to study these phenomena. On the other hand, these inhibitors provide only partial explanations in that while they inhibit CA enzymatic activity all other functions can remain unaffected. Based on these facts, it will still be challenging to completely understand the role of each CA isozyme, although the major pieces in this puzzle have now been identified in the gastrointestinal tract organs.

## Methods

### RNA extraction and first strand cDNA synthesis

The tissue samples used for mRNA quantification were obtained from 10-week-old NMRI mice (four males and four females) with the approval of the Animal Care Committee of the University of Tampere. The tissues extracted included the pancreas, liver, oesophagus, stomach, duodenum, jejunum, ileum, and colon. All specimens used for quantitative real-time PCR were snap frozen upon extraction and stored at -80°C until use. Total RNA was isolated from tissue samples using TRIZOL reagent (Invitrogen, Carlsbad, CA) following the manufacturer's instructions. After digestion of potential residual DNA with RNase-free DNase I (Novagen, Madison, USA), the resulting RNA samples were further purified with phenol/chloroform followed by precipitation with ice-cold ethanol. RNA concentration and purity was determined by optical density measurement at 260 and 280 nm. RNAs extracted from males and females were separately pooled to reduce the potential for individual variation. 3 μg of total RNA was converted into first strand cDNA using the First Strand cDNA Synthesis kit (Fermentas, Burlington, Canada) and random hexamer primers according to the protocol recommended by the manufacturer.

### Quantitative real-time PCR

The amount of mouse Car1, Car2, Car3, Car4, Car5a, Car5b, Car6, Car7, Car9, Car12, Car13, Car14, and Car15 transcripts in different tissues was assessed by quantitative real-time PCR using the Lightcycler detection system (Roche, Rotkreuz, Switzerland). Real-time PCR primers were designed on the basis of the complete cDNA sequences deposited in GenBank (accession numbers: NM_009799 for Car1, NM_009801 for Car2, NM_007606 for Car3, NM_007607 for Car4, NM_007608 for Car5a, NM_019513 for Car5b, NM_009802 for Car6, NM_053070 for Car7, NM_139305 for Car9, NM_178396 for Car12, NM_024495 for Car13, NM_011797 for Car14, and NM_030558 for Car15). To avoid amplification of contaminating genomic DNA, two primers from each primer set were placed in different exons. *GAPDH *(glyseraldehyde-3-phosphate dehydrogenase), *HPRT1 *(hypoxanthine phosphoribosyltransferase 1), and *SDHA *(succinate dehydrogenase complex, subunit A, flavoprotein (Fp)) were used as internal RNA controls to normalize the RNA samples for differences. *HPRT1 *primer sequences are available in the public QPPD database [34] with identification number 10050 and the primer sequences for *SDHA *were obtained from the public PrimerBank database [35] with the identification number 15030102a2. *GAPDH *primers were designed on the basis of the complete *GAPDH *cDNA sequences (accession numbers: NM_001001303). Table 1 shows the primer sequences used for *GAPDH *and the 13 CAs.

Every PCR was performed in a total reaction volume of 20 μl containing 0.5 μl of first strand cDNA, 1× of QuantiTect SYBR Green PCR Master Mix (Qiagen, Hilden, Germany), and 0.5 μM of each primer. Amplification and detection were carried out as follows. After an initial 15-min activation step at 95°C, amplification was performed in a three-step cycling procedure: denaturation at 95°C, 15s, ramp rate 20°C/s; annealing at Tm, 20s, ramp rate 20°C/s; and elongation at 72°C, 20s, ramp rate 20°C/s for 45 cycles, and final cooling step. The melting curve analysis was always performed after the amplification to check PCR specificity. To quantify the concentration of reference genes and the 13 Car transcripts in the eight tissues of this study, a standard curve for each gene was established using five-fold serial dilutions of known concentrations of purified PCR products generated from the same primer sets. Each cDNA sample was tested in duplicate, and the crossing point (Cp) value obtained allowed us to determine the amount of the starting message using the specific standard curve. The geometric mean of the three reference genes was used as an accurate normalization factor for gene expression levels [36]. The final relative mRNA expression was indicated as the copy number of target gene in each tissue divided by the corresponding normalization factor and subsequently multiplied by 10^3^. The mean coefficient of variation in Car mRNA levels calculated from duplicate samples was 6.965%.

## Abbreviations

CA, carbonic anhydrase; CA-RP, carbonic anhydrase-related proteins; RPTP, receptor-type protein-tyrosine phosphatases; RT-PCR, reverse transcription-polymerase chain reaction; *GAPDH*, glyseraldehyde-3-phosphate dehydrogenase; *HPRT1*, hypoxanthine phosphoribosyltransferase 1; *SDHA*, succinate dehydrogenase complex, subunit A, flavoprotein (Fp).

## Authors' contributions

PWP carried out the first strand cDNA synthesis and the QPCR experiments and was the primary author of the manuscript. AR extracted total RNA samples used in this study. SP supervised the study design. All authors participated in the revising of the manuscript.

## Figures and Tables

**Table 1:** Primer sequences for quantitative real-time PCR

**Gene**	**Forward primer (5'-3')**	**Reverse primer (5'-3')**	**PCR product size (bp)**	**Tm (°C)**
GAPDH	ATGGTGAAGGTCGGTGTG	CATTCTCGGCCTTGACTG	186	56
Car1	TTGATGACAGTAGCAACC	CCAGTGAACTAAGTGAAG	161	51
Car2	CAAGCACAACGGACCAGA	ATGAGCAGAGGCTGTAGG	122	56
Car3	GCTCTGCTAAGACCATCC	ATTGGCGAAGTCGGTAGG	160	54
Car4	CTCCTTCTTGCTCTGCTG	GACTGCTGATTCTCCTTA	145	55
Car5a	ACCAAAGCAAGGGCATACAG	TGGCACAGAGAAGTCCCACA	104	58
Car5b	AATGGCTTGGCTGTGATAGG	GGCGTAGTGAGAGACCCAGA	187	60
Car6	AAGATTGACGAGTATGCC	TAGGTGTAATAGTGGTGG	145	54
Car7	CAATGACAGTGATGACAGAA	TCCAGTGAACCAGATGTAG	160	55
Car9	CTGAAGACAGGATGGAGAAG	GCAGAGTGCGGCAGAATG	221	57
Car12	CCTATGTTGGTCCTGCTG	CGTTGTAACCTTGGAACTG	143	53
Car13	AATACGACTCCTCACTCC	TGCCGCAACCTGTAGTTC	116	52
Car14	TGTTGTTCTTCGCTCTCCTG	CACTGTCTGTCTGGATATTG	161	53
Car15	AGCACAGCCTGGATGAGA	CAGACACAATGGCAGAGA	170	55

Tm: annealing temperature
